# A pilot study to evaluate incorporating eye care for children into reproductive and child health services in Dar-es-Salaam, Tanzania: a historical comparison study

**DOI:** 10.1186/1472-6955-13-15

**Published:** 2014-06-02

**Authors:** Milka Madaha Mafwiri, Rodrick Kisenge, Clare Elizabeth Gilbert

**Affiliations:** 1 Department of Ophthalmology, Muhimbili University of Health and Allied Sciences, P.O. Box 65001, Dar-es-Salaam, Tanzania; 2 Department of Pediatrics and Child Health, Muhimbili University of Health and Allied Sciences, Dar-es-Salaam, Tanzania; 3 Department of Clinical Research, London School of Hygiene & Tropical Medicine, Keppel Street, London WC1E 7HT, UK

**Keywords:** Child eye health, Primary eye care, Reproductive and child health clinics, Tanzania

## Abstract

**Background:**

Many blinding eye conditions of childhood are preventable or treatable, particularly in developing countries. However, primary eye care (PEC) for children is poorly developed, leading to unnecessary visual loss. Activities for control by health workers entail interventions for systemic conditions (measles, vitamin A deficiency), identification and referral of children with sight threatening conditions and health education for caregivers. This pilot study evaluated integrating a package of activities to promote child eye health into Reproductive and Child Health (RCH) services in Dar-es-Salaam, Tanzania.

**Methods:**

Design: historical comparison study. Fifteen Clinical Officers and 15 nurses in 15 randomly selected RCH clinics were trained in PEC for children in July 2010. They were given educational materials (poster and manual) and their supervisors were orientated. Knowledge and practices were assessed before and 3 weeks after training. One year later their knowledge and practices were compared with a different group of 15 Clinical Officers and 15 nurses who had not been trained.

**Results:**

Before training staff had insufficient knowledge to identify, treat and refer children with eye diseases, even conjunctivitis. Some recommended harmful practices or did not know that cataract requires urgent referral. Eye examination, vitamin A supplementation of mothers after delivery and cleaning the eyes at birth with instillation of antibiotics (Crede’s prophylaxis) were not routine, and there were no eye-specific educational materials.

Three weeks after training several clinics delivering babies started Crede’s prophylaxis, vitamin A supplementation of women after delivery increased from 83.7% to 100%, and all staff included eye conditions in health education sessions. At one year, trained staff were more likely to correctly describe, diagnose and treat conjunctivitis (z=2.34, p=0.04)(30%-vs-60.7%). Mystery mothers observed health education sessions in 7/10 RCH clinics with trained staff, five (71.4%) of which included eye conditions.

**Conclusions:**

Primary eye care for children in Dar-es-Salaam is inadequate but training RCH staff can improve knowledge in the short term and change practices. Attendance by mothers and their children is high in RCH clinics, making them ideal for delivery of PEC. Ongoing supportive supervision is required to maintain knowledge and practices, as well as systems to track referrals.

## Background

Many eye diseases of childhood in developing countries can be prevented (e.g. corneal scarring from measles and vitamin A deficiency disorders (VADD), or treated (e.g. cataract) so preventing visual impairment or blindness [1]. In 2002, the World Health Organization (WHO)/Lions Sight First project for the prevention of blindness in children identified 10 key activities which could be implemented by primary health workers to promote the eye health of children in sub-Saharan Africa and South East Asia [2]. Five of the activities relate to the control of conditions which can be associated with visual loss (e.g. measles, VADD) while the other five focus on specific eye conditions (e.g. conjunctivitis of the newborn, cataract) (Table 1) and may also cause visual loss. Some of these interventions have a strong evidence of efficacy [3] whereas there is less evidence for others [4]. One of the activities, vitamin A supplementation of women immediately after delivery, although not a Ministry of Health policy, is being practiced. The WHO/Lions Sight First project for prevention of blindness in children recommended that the 10 key activities be integrated into existing primary or community health care services, such as Reproductive and Child Health (RCH) clinics, the Expanded Program of Immunization, the Integrated Management of Childhood Illnesses (IMCI), and nutrition and vitamin A supplementation programmes. However, to our knowledge the 10 key activities have not been integrated into existing primary or community health care services in any country.

**Table 1:** Ten key activities to promote healthy eyes in children

**Ocular condition**	**Activity**
**Control of conditions which can be associated with visual loss**	
Vitamin A deficiency	1. Give vitamin A supplements to children routinely
	2. Give vitamin A supplements to mothers after delivery
	3. Promote breast feeding and good nutrition
Measles	4 Give vitamin A supplements to children with measles or malnutrition
	5. Immunize children against measles
**Control of ocular conditions**	
Conjunctivitis of the newborn	6. Clean the eyes of babies at delivery and apply antibiotic eye drops
Trachoma	7. Keep children’s’ faces clean
Cataract	8. Refer children with poor vision or white pupils to an eye worker
Traditional eye remedies	9. Avoid the use of traditional eye medicines
Trauma	10. Refer children with history of injury to an eye worker

In Tanzania, dispensaries and health centres are responsible for managing common illnesses and identification and referral of those requiring secondary or tertiary care. These health facilities are staffed by Clinical Officers (COs) who are trained for 2–3 years after secondary school education. They are also staffed by registered and enrolled nurses (more than 3 years nurse training) and public health nurses (trained for 1 year). Registered and enrolled nurses are the main providers of antenatal care and deliveries. All primary, secondary and tertiary level facilities provide RCH services for family planning, safe motherhood, child survival, and control of sexually transmitted diseases and HIV/AIDS. More than 90% of mothers attend antenatal and postnatal care and approximately 50% of deliveries take place in these facilities [5]. Aspects of RCH involving child health are based on the Integrated Management of Childhood Illness (IMCI) which gives guidance on how to assess children aged 2 months to 5 years, how to classify the severity of the condition, and the treatment required.

Despite frequent contact with trained health personnel from birth to five years, children with eye conditions usually present very late to tertiary eye departments in low and middle income countries, including Tanzania [6-10]. Reasons include animist or fatalistic beliefs about causation, lack of awareness that the condition is treatable, poor levels of education amongst mothers, fear of surgery and indirect and direct costs. Health workers sometimes also give the wrong advice as they too are unaware of the correct management and the urgency required. The consequences of late management of congenital cataract are considerable, impacting on the family as well as the child [11] as delayed surgery can lead to poor visual outcomes [7,9,10] and psychosocial and physical developmental delay [12-15]. Delay in the diagnosis and management of other eye conditions can have other consequences. For example, delay in identification and treatment of retinoblastoma can lead to death of the child.

The purpose of this pilot study was to evaluate implementation of 10 key activities for healthy eyes by primary level staff in RCH clinics in Dar-es-Salaam, focusing on changes in their knowledge, attitudes and practices following training, and whether change was maintained over 12 months.

## Methods

### Overview

This study was a historical comparison study. All public RCH clinics in the three districts of Dar-es-Salaam, Tanzania were enumerated, followed by simple random sampling to select 15 RCH clinics so that the number selected reflected the population of each district. In each RCH facility, one CO and one nurse providing RCH services were randomly selected for interview and training. Participants in selected RCH clinics were trained in the 10 key activities in July 2010 and were given educational materials for themselves and their clinic. Three weeks later they were re-interviewed, to assess change in their knowledge of eye disease in children or practices. One year later they were visited again, to assess whether they had maintained their knowledge and practices. Findings at one year were compared with a different group of RCH staff who had not been trained.

### Pre-intervention phase

#### Development of educational materials

An illustrated reference manual was written for RCH staff which covered simple anatomy of the eye and descriptions of each of the 10 key activities and their importance in preventing eye diseases and visual loss. The manual included treatment and referral guidelines and the location and telephone number of the two tertiary eye departments in Dar-es-Salaam. A poster was also developed, with simple text in Swahili. The manual and poster included photographs of African women and children to illustrate each activity.

A semi-structured questionnaire for use before and after training was developed to collect demographic and professional data, activities performed, workload and the challenges RCH staff faced in their work. Knowledge of eye diseases and how they would manage them were assessed through questioning, and by images of children with conjunctivitis in the newborn, cataract and trauma.

#### Review of basic training curricular of RCH staff

The training curricular for RCH staff were requested from the Directorate of Human Resource of the Ministry of Health and Social Welfare to assess whether primary eye care for children was included.

#### Observation of RCH activities

Activities performed by RCH staff while at work were observed and recorded using a checklist. Data on the number and diagnoses of patients referred for further management was obtained from registers. The type, content, use, and display of educational materials were noted.

### Intervention phase

#### Study population and sampling

All government primary health facilities providing RCH services for children from each district of Dar-es-Salaam region were enumerated and assigned a number. Each number was written on a separate piece of paper, and then fifteen papers representing 15 facilities were randomly selected. Selected facilities included 6 health centres and 9 dispensaries. The total number selected in each district was proportionate to the population of the district such that 6 RCH clinics were selected in the largest district, Kinondoni, 5 from Temeke and 4 from Ilala district. In each RCH clinic one nurse and one CO were selected; if there were two or more nurses, one was selected at random. Two district RCH coordinators, who supervise staff in the clinics, were also oriented towards the intervention so they could provide supportive supervision. A year later, in July 2011 a further 15 facilities were selected from the same districts, using the same methods as in 2010.

#### Training of clinical offices and nurses

In July 2010, 30 staff underwent one day intensive training (15 nurses, 15 COs) at Muhimbili National Hospital (MNH). Topics included simple anatomy of the eye; the causes, treatment and prevention of common eye conditions and the causes of blindness in children. Emphasis was placed on how to identify, treat or refer each condition. Each of the 10 key activities and its role in the prevention of blindness was discussed. Teaching methods included lectures, and group discussion. On the same day, Participants visited the pediatrics eye unit at MNH to observe visual acuity testing, refraction and low vision assessment. They were shown children with corneal scars, congenital and developmental cataracts, and retinoblastoma.

Participants were asked to refer all children with abnormal eyes, poor vision, squint (non-aligned eyes), white pupils (from cataract or retinoblastoma) to Mnazi Mmoja (MM) Health Centre where two Assistant Medical Officers in Ophthalmology (AMO-O) trained in child eye health screened and referred the children to tertiary care, as is usual practice in Tanzania. Participants were requested to keep records of referrals and contact the Childhood Blindness Coordinator at MNH if they suspected a child to be blind. The coordinator would then record details of the child and follow up with the mother and/or with staff at MNH to find out whether the child attended for assessment. Each participant was given a poster, training manual and a torch for examining the front of the eye.

Participants were also interviewed in depth to find out what they knew about beliefs and behavior in the community in relation to eye diseases affecting children, and traditional remedies used to treat eye diseases. RCH Coordinators were also interviewed to triangulate what RCH staff reported and to explore how challenges in implementing the 10 key activities could be overcome.

The two RCH coordinators were asked to clarify/confirm that they had responded to issues raised by the RCH workers e.g. in relation to their daily duties, or who they should refer children to, or how they should prioritize their workload.

### Post-intervention phase

#### Data collection one year after training (i.e. in July 2011)

All clinics with staff trained in 2010 were visited in July 2011 for re-interview using the same questionnaire as at baseline. Two staff not present were interviewed by telephone. The following were observed: whether the poster was displayed and the manuals and torch were still available, and details of referral. Staff were asked whether they used the poster and manual. The MM Health Centre was visited, to ascertain the number of children referred with eye conditions in the 6 months before and after the 2010 training.

In July 2011, 20 mystery mothers were selected at random from the RCH clinics at MM Health Centre and Temeke hospital. Those agreeing to participate were assigned one RCH clinic to visit from the 10 control clinics and 10 clinics where staff had been trained a year earlier. They were asked to attend the health education session and report back on the topics taught. They were also asked to tell staff that a child at home had white spots in his eyes and to report back the advice she was given.

After visits by the mystery mothers, 30 RCH staff in the 15 control clinics were trained using the same materials and methods as in 2010. The same questionnaire was administered and data from the untrained (control) group was compared with the 2010 trained group.

### Data management

Quantitative data were analyzed using the statistical package for social sciences (SPSS) version 15.00. Descriptive statistics were depicted using absolute numbers and simple percentages. Chi square and t-significance tests were used where appropriate to compare differences in knowledge and practices between the trained and untrained groups.

Ethical approval was obtained from the Research and Ethics Committees of the London School of Hygiene & Tropical Medicine and Muhimbili University of Health and Allied Sciences. Written informed consent was obtained from all participants.

## Results

### Training curricular for RCH staff and educational materials

The curriculum for the basic training of RCH staff did not include eye diseases in children, apart from the prevention and treatment of ophthalmia neonatorum. IMCI only refers to eye conditions occasionally e.g., that conjunctivitis can complicate measles. All RCH clinics had educational materials, mainly posters on a range of topics. Apart from prevention of ophthalmia neonatorum, none were specifically on eye conditions.

### Study population

In 2010, two participants were not available for the three week post-training interview. In 2011, four were not available: two could not be contacted and two had transferred to another facility.

### 2010: three week follow up

Three weeks after training demonstrated that there had been considerable improvement in knowledge, and change in some practices. For example, 24 (85.7%) and 25 (89.2%) RCH staff could recognize conjunctivitis and cataract from images after training compared with 14 (46.7%) and 2 (6.7%) respectively before training. Staff in 4 RCH facilities conducting deliveries had initiated Crede’s prophylaxis and all RCH staff reported delivering eye health education compared with 66.6% before training. All staff had put up their posters and had their manuals at work. There was also change in attitudes as trained RCH staff started to train other workers on child eye health using the poster and manual. The poster and manual were also used during health education sessions, to illustrate different aspects of eye health to mothers, and for reference. All staff thought the poster was more useful than the manual.

“*We have displayed our poster at the outpatient department entrance and it is getting a lot of attention from patients and staff alike. Distribute it to all RCH clinics*”.

“*Nowadays we use this poster to illustrate eye conditions during health education*”.

“*Patients crowd around it*, *reading and asking us questions. They show a lot of concern about their children. They compare the appearances on the pictures and ask questions about themselves and their children*”.

When asked why the poster had attracted so much attention, one clinical officer remarked:

“……. *probably because the pictures are real as opposed to drawings*”.

The following are quotes from RCH staff in relation to the manual:

“*This simple manual is good for reference*.....*First we look at the poster then consult the manual for further information*”.

“….*when we came back from training*, *our colleagues wanted to know everything* ……. *All seven photocopied the manual for their reference*”.

When asked whether the training had improved their knowledge of child eye health, one CO remarked:

“*The training has been very beneficial. For me*, *I used to feel very incompetent when a patient with an eye condition came to the clinic. I just referred. Now I know and I am able to give medicine or refer as required*……… *But it was too short. May be one week would be better*…”.

The RCH coordinator in one district commented that there had been a change in some practices in relation to eye care:

“*In this RCH*, *whenever we see a child with eye problems*, *we refer to* {*staff name*} (*CO*) *because he had the training*”.

Participants expressed appreciation for the training manual and poster:

“*I look at the pictures and look at the patient then I know the condition*…”.

In 2010, twenty one of the 30 untrained RCH staff reported the following beliefs in the community: spicy food during pregnancy causes red discharging eyes 11(36.6%), and eating clay 2 (6.7%) or eggs 1 (3.3%) during pregnancy causes blindness in children. Other causes included angering ancestors 2 (6.7%), a child looking at his/her naked parents 2 (6.7%) and witchcraft 1 (3.3%). Other reported beliefs were: painful swellings on eyelids of children (sties) were due to misbehavior of the mother towards her in-laws 2 (6.7%). Nine (30%) respondents did not know of any misconceptions. Local herbs (pounded, chewed, tree sap) and milk were the commonest home remedies for red eyes.

Reported barriers in identification and referral of children cited by untrained RCH staff included: inadequate knowledge and skills in child eye health, lack of diagnostic tools and drugs, lack of supervision, unclear roles and responsibilities, long distances to referral centers, and overwork, as shown by the following quotes:

“*I have never been trained in eyes*….*We did not have an eye clinic at my hospital*”.

*(Even though we had some teaching on eye diseases*, *we have had no refresher training*-------*eye doctors should do like IMCI. IMCI*…..*send workers for refresher training)*.

“*We have nothing to examine eyes with*, *I have seen a chart to examine eyes*, *we do not have one*…….*drugs to use in new born babies*…”.

“*It is only you who have visited us*….. *I have never seen an eye doctor visit us before. We don*’*t know or learn on eyes. If you want the eye program to continue*, *you should do what the HIV and TB doctors do. They visit the health centers and dispensaries regularly* (*CO*)……”.

“*Honestly*, *we used to clean and instill eye drops in new born babies. I do not know how and why we stopped*. …….*there is nobody reminding them*, *nobody follows up*…. *Probably just laziness* (*RCH coordinator*)”.

“*It is very sad to refer a patient who will not go for referral because sometimes it is too far* (*nurse*)”.

“*We are overburdened with work. Usually there is one enrolled nurse per shift and sometimes two or more mothers deliver within minutes of each other*.. *You have to concentrate on making sure they deliver safely and pay minimal attention onto issues like cleaning eyes* (*a nurse who used to work in a district hospital*)….”.

### 2011 evaluation to compare trained and control staff

#### Characteristics of trained and untrained staff at the 2011 evaluation

Equal numbers of COs and nurses in the trained and control group took part in the study (i.e. 15 in each group) (Table 2) at both time points. Clinical Officers were more likely to be male than nurses in both groups (Z = 2.15, p = 0.03 trained group; Z = 2.19, p = 0.02 control group). The mean age of staff in the control group was 45.3 years compared with 42.9 years for those trained (Z = 0.79, p = 0.42). The mean number of years staff had worked in the RCH clinics was 5.6 and 4.0 years for the control and trained groups respectively but the difference was not statistically significant. COs in both groups were younger than nurses and had been in the post for a shorter period of time, but differences were not significant. The workload was similar between RCH clinics with trained and control staff.

**Table 2:** Demographic and professional characteristics of RCH staff who were trained (n = 30) and not trained (n = 30)

**Characteristic**		**Staff in control RCHs N** = **30**			**Staff in trained RCHs N** = **30**			**P-value**
		**COs (n = 15)**	**Nurses (n = 15)**	**All (%)**	**COs (n = 14)**	**Nurses (n = 14)**	**All (%)**	
		**N**	**N**	**N (%)**	**N**	**N**	**N (%)**	
Age group	26-35	3	2	5 (16.6)	6	0	6 (20)	
	36-45	4	6	10 (33.3)	6	6	12 (40)	
	46-55	8	7	15 (50)	3	9	12 (40)	
	Mean, SD	45.4 (8.5)	45.4 (7.6)	45.3 (7.9)	38.3 (8.1)	47.5 (6.7)	42.9 (8.7)	0.4
Duration at RCH (yrs)	1-3	7	4	11 (36.6)	13	5	18 (60)	
	4-6	4	6	10 (33.3)	2	5	18 (60)	
	>6	4	5	9 (30.0)	0	5	5 (16.6)	
	Mean, SD	5.0 (4.6)	6.3 (5.5)	5.6 (5.1)	3.1 (1.4)	4.9 (2.4)	4.0 (2.1)	0.6
Sex	Male	6	0	6	7	0	7	
	Female	9	15	24	8	15	23	
Highest qualification (yr)	1979-89	3	6	9 (30)	2	2	4 (13.3)	
	1990-99	2	2	4 (13.3)	4	7	11 (36.6)	
	2000-09	10	7	17 (56.6)	9	6	15 (50.0)	0.3
Previous training*	Yes	11	12	23 (76.7)	13.	12	23 (76.7)	
	No	4	3	7 (23.3)	2.	3	5 (23.3)	
Refresher training*	Yes	0	0	0	0	0	0	
	No	15	15	30 (100)	15	15	30 (100)	
**Number of children seen each day**								
		COs	Nurses	Register	COs	Nurses	Register	
(Mean, SD)		30 (17.8)	89 (48.6)	184 (73.5)	35 (16.1)	86 (48.2)	178 (69.2)	

*CO* = Clinical Officer; * = in eye-care.

Almost 80% of participants in both groups had received some primary eye care training during their basic professional training, but none had received refresher training. Both RCH coordinators were females and their ages were 45 and 49 years. The mean age of mystery mothers was 32 years.

All RCH facilities monitored the weight of children, undertook immunization with vitamin A supplementation, which are recorded on the RCH card and gave health education (Table 3). However, most clinics did not routinely examine the eyes of children, nor were all mothers supplemented with vitamin A immediately after delivery. None of the RCH clinics had torches for eye examination. Three weeks after training, all (100%) retained and used their torches while 9/28 (23.1%) had torches at one year. Three of those who had torches at one year had purchased them as personal property after the ones issued at the training broke down or were lost.

**Table 3:** Daily activities of RCH staff

**Activity**	**Facilities where applicable**	**RCH clinics with control staff (n = 30)**		**RCH clinics with trained staff (n = 28)**	
Daily activities performed at RCH					
Weight monitoring	30	100%		100%	
Immunization	30	100%		100%	
Health education	30	100%		100%	
Vitamin A supplementation – children	30	100%		100%	
Vitamin A supplementation – mothers	30	86.7%		100%	
Eye exam	30	33.3%		100%	
Children with eye problems seen/week		N	%	N	%
None		4	13.3	1	3.5
One or more		26	86.5	27	96.3

From a review of the MM Health Centre register, 108 (18 per month) children had been referred for tertiary eye care in the 6 months before training which increased to 135 (22 per month) in the 6 months after training. It was not possible to determine how many had attended tertiary facilities nor ascertain the diagnosis.

#### Experience and knowledge of eye diseases in children

Purulent discharge was the commonest condition seen by both groups (Table 4). Staff who had been trained were more likely to make the correct diagnosis and management decisions than staff who had not been trained. Three control COs said they would treat conjunctivitis with steroid eye drops.

**Table 4:** Conditions seen and management decisions

	**Control (n = 30)**			**Trained (n = 28)**			**P-value**
**Eye condition**	**COs (n = 15)**	**Nurses (n = 15)**	**All (%)**	**COs (n = 14)**	**Nurses (n = 14)**	**All (%)**	
Purulent discharge	10	11	21 (70)	10	9	19 (67.8)	0.14
Others	5	2	7 (23.3)	4	5	9 (32.1)	
Did not remember		2	2 (6.6)			0	
**Management of purulent discharge:**							
Described symptoms did not know diagnosis	3	5	8 (26.6)	0	2	2 (7.1)	0.16
Described symptoms but wrong diagnosis	2	2	4 (13.3)	0	0	0	
Described symptoms with correct diagnosis and treatment	5	4	9 (30)	10	7	17 (60.7)	0.04
Did not remember the child they last saw	0	2	2 (6.6)	0	0	0	
Other actions	5	2	7 (23.3)	4	5	9 (32.1)	

COs = Clinical Officer.

Trained staff could name more eye conditions of childhood than untrained staff (mean 3.2 +/-1.3; vs mean 1 +/- 1.0; z = 4.610, p < 0.0001). In both groups COs were more knowledgeable than nurses, with the difference being statistically significant for trained COs (t = 2.25, p < 0.02). Trained staff were also able to name more management options than untrained staff (2.16 +/- 1.2 vs 1.3 +/- 1.0; z = 2.34, p < 0.01). The mean number of management options known by untrained COs was higher than among untrained nurses but differences were not statistically significant (1.6 +/- 0.95 vs 1.3 +/- 0.9 respectively; t = 0.80, p < 0.2).

Only ten control RCH staff could correctly identify conjunctivitis from an image compared with 23 trained staff (33% vs 82.1%, z = 4.25, p < 0.001) (Table 5). Twenty five control staff did not know or misdiagnosed cataract compared with 11 trained staff (83.3% vs 39.3%, z = 3.45, p < 0.001). Management decisions for cataract and trauma were also significantly better among trained staff. Indeed, three control participants mentioned that cataract in children should be treated with vitamin A.

**Table 5:** Diagnostics skills and management decisions of trained and control RCH staff

**Identification**	**Control (n = 30)**			**Trained (n = 28)**			**P-value**
	**COs (n = 15)**	**Nurses (n = 15)**	**All (%)**	**COs (n = 14)**	**Nurses (n = 14)**	**All (%)**	
**Image of conjunctivitis:**							
Correctly identified	6	4	10 (33.3)	13	10	23 (82.1)	0.00
**Image of cataract:**							
Correctly identified	3	2	5 (16.6)	9	8	17 (60.7)	0.01
**Management of cataract:**							
Refer for surgery	3	3	6 (20.0)	10	11	21 (70.0)	0.00
Refer, did not know treatment	6	7	13 (43.3)	5	3	8 (28.5)	0.4
Management not known	6	5	11 (36.6)	0	0	0 (0.0)	0.00
**Management of trauma:**							
Immediate referral	2	1	3 (10.0)	7	8	15 (53.5)	0.00

#### Practices in relation to prevention

Seventeen RCH clinics had facilities for normal deliveries (10 with trained staff; 7 with control staff). Only 28.5% of control clinics were routinely performing Crede’s ocular prophylaxis (i.e. cleaning the eyes immediately after birth and instilling antibiotic eye drops) compared with 57.1% of clinics with trained staff. In control clinics some cleaned the eyes only while others instilled eye drops, but only if the mother had a sexually transmitted disease. Reasons for not performing Crede’s prophylaxis were lack of supervision, being overworked and lack of eye drops.

Training reinforced by the educational materials led to change of practice.

“*After training*, *we conducted a clinical meeting and taught our colleagues what we had learnt. Honestly*, *we were not putting eye drops in newborn eyes. The practice had been forgotten*….. *Now we have set out a protocol to have antibiotic eye drops as one of the components of a delivery tray*”.

#### Health education conducted in relation to the eye

Two health education methods were used: health talks by nurses three-four times a week to groups of mothers in the waiting area, and one to one counseling of mothers by nurses and COs. Nurses prepared a timetable of topics for the health talks, which were changed if there was a disease outbreak (e.g. measles). Almost half of untrained staff (43.3%) had never given health education on eye conditions but over three quarters of trained staff had held a session on eye conditions within a week of the evaluation visit (Table 6). There were also considerable differences in the topics covered, with trained staff being more likely to cover topics specifically related to eye conditions than untrained staff. Mystery mothers visited and reported that they had observed health education sessions in 15 out of 20 RCH clinics. Health education related to the eye was observed in 0/8 (0%) and 5/7 (71.4%) RCH clinics with untrained and trained staff respectively (Table 7).

**Table 6:** Health education relating to the eye delivered by RCH staff

	**Control (n = 30)**			**Trained (n = 30)**			
	**COs (n = 15)**	**Nurses (n = 15)**	**All (%)**	**COs (n = 14)**	**Nurses (n = 14)**	**All (%)**	**P-value**
**When last eye health education was delivered:**							
Within last 7 days	5	5	10 (33.3)	11	11	22 (78.5)	<0.001
Within last 4 weeks	4	2	6 (20.0)	3	3	6 (21.4)	0.4
Within last 1 year	1	0	1 (3.3)	0	0	0	
Never	5	8	13 (43.3)	0	0	0	
**Content of health education:**							
*Not specifically related to eye health*							
Facial hygiene	6	3	9 (30)	4	1	5 (16.6)	
Delivery	1	2	3 (10)	0	0	0	
Infection during delivery	2	2	4 (13.3)	0	0	0	
Examine baby after delivery	0	0	0	0	1	1 (3.5)	
*Specifically related to eye health*							
Eye diseases	1	0	1 (3.3)	2	0	2 (7.1)	
Importance of immunization	0	0	0	0	3	3 (10.7)	
Importance of vitamin A	0	0	0	3	6	9 (32.1)	
Identification referral of those with eye disease	0	0	0	4	3	7 (25)	
Do not use non-prescribed/traditional eye medicine	0			1	0	1 (3.5)	
**Total**	15	15	30 (100)	14	14	28 (100)	

**Table 7:** Mystery mothers report on health education delivered by RCH staff

**Topics covered**	**Control n = 10**		**Trained n = 10**	
	**N**	**%**	**N**	**%**
**Not specifically related to eye health:**				
Safe delivery	2	20	1	10
Sexually transmitted diseases	1	10	1	10
Lactation and good nutrition	1	10	0	0
Measles and immunization	4	40	0	0
**Specifically related to eye health:**				
Vitamin A and the eye	0	0	1	10
Measles immunization and the eye	0	0	3	30
Identification of children with eye diseases	0	0	1	10
**None observed:**				
No health education session	1	10	2	20
Arrived too late for health education session	1	10	1	10

Trained nurses made the following comments:

“*These days*, *we have scheduled eye disease topics on the health education timetable*”.

“*I used to counsel mothers to exclusively breast feed* …., *but I did not know that it prevents eye diseases*.….*now I feel more confident talking to mothers about it*”.

Enthusiasm for eye health education was shown by a nurse who had visited a trachoma endemic area:

“*These days I frequently see adults with destroyed corneas and in*- *turned eyelashes just like on the pictures*! ….*before the training*, *I never knew what was wrong with all these people. Oh*, *I feel like calling a big meeting to give a lecture*…. *I advise them to go to hospital*”.

Mystery mothers reported that measles and measles immunization were the topics most frequently covered in health education sessions (7/15 sessions) (Table 7), but only trained nurses said that measles can affect the eye and that measles immunization can prevent blindness. Trained staff also gave talks on vitamin A and the eye and on other eye conditions in children, unlike untrained staff. All mystery mothers reported that RCH staff had instructed them to bring the affected child to the clinic for examination.

## Discussion

Findings from this pilot study show that knowledge about eye conditions in children and how they can be prevented or managed was low among RCH staff in clinics in Dar-es-Salaam, even for common conditions such as conjunctivitis. Indeed, some staff suggested inappropriate treatments, such as steroid drops for conjunctivitis or vitamin A capsules for cataract. This inappropriate treatments are likely to exacerbate the condition, or lead to delay in accessing appropriate care, which in the case of cataract, increases the risk of poor visual outcomes [9,10,16]. The relatively low level of knowledge almost certainly reflects the limited exposure staff would have had to primary eye care during their initial training. Also as noted in the results, preventing ophthalmia neonatorum is the only eye-specific condition included in the initial curriculum. The lack of educational materials on eye conditions in the clinics also suggests that eye care had not been a topic covered in training or continuing professional development. Our findings are in contrast to a study in Ethiopia where primary health care workers were more knowledgeable about the causes of blindness in children [17] probably because of the on the job training they had received.

Staff in the study were eager to learn and training improved their knowledge and management decisions, as in a study in India [18]. It is important to note that knowledge was better at three weeks than at one year after training. This emphasizes the need for supportive supervision, which should focus on continuing professional development and not just administrative or managerial matters [19]. Studies elsewhere have shown that training alone can give disappointing results in terms of changing practices and behaviours [20]. A future study will need to explore barriers which may limit the ability of RCH staff to change their practices e.g. lack of autonomy in decision making, unclear roles and responsibilities, or lack of time, so that these barriers can be addressed [21].

In our study COs were generally more knowledgeable than nurses, and could name more eye conditions and their management than nurses before training, reflecting their different educational backgrounds and roles. However, some COs recommended some potentially harmful treatments, such as treating conjunctivitis with steroids. Although COs diagnose and treat conditions, nurses are the first point of contact with mothers and their children, and provide the health education. Although the study did not explore the satisfaction of RCH workers with the training methods, duration and training materials in any depth, some participants thought the training was too short. Future studies should explore the optimal duration of training for different cadres of RCH staff considering that they have different educational backgrounds. Most staff were enthusiastic about the training materials but any further studies should explore whether they can be improved.

### Practice of RCH staff

Staff were already providing a good service in relation to measles immunization and vitamin supplementation but they did not know that these interventions also reduce blindness in children. The high coverage of these interventions, which reflects the priorities and policies of the Ministry of Health, are reducing under 5 mortality rates in Tanzania. There is also anecdotal evidence of less corneal blindness in children [22]. However, activities specific to eye health, such as Crede’s prophylaxis, were not being routinely performed. Reasons for not using Crede’s prophylaxis included overwork, and lack of supervision and eye drops. However, after training half of the facilities that conducted deliveries resumed the practice, which suggests that improved knowledge in relation to the importance of ophthalmia neonatorum motivated staff to change their practices, which were maintained at one year. The number of children referred to MM Health centre increased after training, but a limitation of this study was that it was not possible to track these children to ascertain how many were referred unnecessarily, nor to determine the accuracy of diagnoses made by RCH staff. Efforts to track referred patients and their diagnoses should be made in a future study.

### Health education

Another positive change was that trained staff felt confident to deliver health education on eye conditions, often including eyes when talking about other conditions, such as measles. However, to be effective, health education should take place alongside improvement in the quality of services, in both clinical and non clinical care [23]. Topical medication and diagnostic equipment, such as torches, must be provided, to enable staff to put into practice what they talk about during health education.

Educational materials for those using health services are a requirement of PEC [24] and the lack of information on eye conditions for mothers as well as staff shows that this aspect of PEC also needs to be addressed. In this study efforts were made to make the poster relevant and attractive, with messages that were easy to understand. Staff reported that mothers responded positively to the poster and they valued and used the poster and manual for reference and health education.

The misconceptions and beliefs in the community about eye conditions reported by staff in this study may partly explain delay in accessing eye services for children. Other studies in Tanzania and Sub-Saharan Africa show that home treatments and remedies, and those recommended by traditional healers, are commonly used for eye conditions in children [25]. Some of these practices can be harmful (e.g., causing trauma, infection) while others can lead to delay in seeking appropriate treatment. It is important that staff in RCH clinics address these topics during health education [26].

## Conclusions

This is the first study to evaluate the 10 key activities for healthy eyes as a package of care since they were recommended over ten years ago. We explored the provision of eye care for infants and children in RCH clinics and investigated providers’ perspectives on barriers to providing PEC in children. This preliminary study shows that mothers and children use the clinics in large numbers, and RCH staff are willing to learn and change their practices. The next phase will be to assess the impact of this intervention, focusing on health seeking behavior and attitudes in the community towards eye care for children which may limit access, and will explore whether PEC in children can become embedded within IMCI. Health management information systems will need to be strengthened so that children referred can be tracked through the health system, and we will discuss with managers how supportive supervision can be improved and relevant supplies provided and maintained. As Dar-es-Salaam is not representative of Tanzania, the study will take place in rural and per-urban areas in another region.

## Abbreviations

PEC: Primary Eye Care; RCH: Reproductive and Child Health; IMCI: Integrated Management of Childhood Illnesses; COs: Clinical Officers; MNH: Muhimbili National Hospital; MM: Mnazi Mmoja Health Centre.

## Competing interests

All authors declare that they have no competing interests.

## Authors’ contributions

MM and CG conceived and designed the study. All data collection and analyses were undertaken by MM. All authors contributed to the manuscript. All authors read and approved the final manuscript.

## Pre-publication history

The pre-publication history for this paper can be accessed here:

http://www.biomedcentral.com/1472-6955/13/15/prepub
